# Profiles of Dyadic Self-care Congruence and Patient Symptom Burden in Heart Failure

**DOI:** 10.1097/JCN.0000000000001239

**Published:** 2025-08-11

**Authors:** Giulia Locatelli, Ercole Vellone, Davide Ausili, Christopher Sean Lee

**Affiliations:** Giulia Locatelli, PhD, RN, School of Medicine and Surgery, University of Milano-Bicocca, Monza, Italy.; Ercole Vellone, PhD, Department of Biomedicine and Prevention, University of Rome Tor Vergata, Italy; and Department of Nursing and Obstetrics, Wroclaw Medical University, Poland.; Davide Ausili, PhD, School of Medicine and Surgery, University of Milano-Bicocca, Monza, Italy.; Christopher Sean Lee, PhD, Boston College William F. Connell School of Nursing, Chestnut Hill, Massachusetts.

**Keywords:** caregivers, heart failure, self-care, symptom burden

## Abstract

**Background:**

Patients with heart failure (HF) experience several symptoms that can be alleviated by self-care, to which caregivers can contribute too. The degree of congruence between patients' and caregivers' perceptions of chronic illness should be explored to gain a holistic understanding of the dyadic illness management.

**Objective:**

The aim of this study was to explore dyads in HF based on the congruence between patient self-care and caregiver contribution to patient self-care, and the levels of physical symptom burden in each dyad type.

**Methods:**

This is a secondary analysis of data from the MOTIVATE-HF randomized controlled trial, which enrolled 510 dyads of patients with HF and their caregivers. Latent class mixture modeling was used to identify distinct patterns among empirical Bayes estimates of within-dyad congruence in self-care maintenance, management, and confidence, as well as symptom burden score. χ^2^ Tests and analysis of variance were used to compare characteristics among the identified classes.

**Results:**

We identified 3 classes of dyads. In class 1 (17%), caregivers contributed more to HF care than patients; patients in this class reported the highest symptom burden. In class 2 (7%), patients performed more self-care than caregivers contributed to HF care; patients in this class reported the lowest symptom burden. In class 3 (76%), patients and caregivers contributed similarly to HF care.

**Conclusions:**

In HF, different dyad types exist based on the congruence between patient self-care and caregiver contribution to self-care, and the levels of patient symptom burden. This is pivotal to help generate an evidence base for symptom management interventions.

**What’s New and Important**

- Different types of dyads exist based on the congruence between patient self-care and caregiver contribution to self-care, and the levels of patient symptom burden.
- In some dyads, patients perform more self-care compared with how much their caregivers contribute to self-care and, in these dyads, patients experience the lowest level of symptom burden.
- In other dyads, caregivers contribute more to self-care compared with how much patients themselves perform self-care and, in these dyads, patients experience the highest level of symptom burden.

Patients with heart failure (HF) experience several symptoms, often occurring simultaneously,^1^ which contribute to a decreased quality of life,^2^ high hospitalization^3^ and mortality rates,^4^ and high symptom burden.^3,5–7^ Symptoms can be alleviated by adequate self-care behaviors, and at the same time, symptoms can also influence the self-care process itself.^8–10^ Self-care is the process of maintaining health and managing illness. Specifically, self-care behaviors include self-care maintenance (eg, taking medication as prescribed to maintain health and prevent symptom exacerbations), self-care monitoring (eg, routine testing to recognize early changes), and self-care management (eg, changing the diet or medication dose based on emerging symptoms to effectively address them).^11^ In addition, self-care self-efficacy (also called self-care confidence) refers to the degree of confidence in performing self-care.^11^

Self-care behaviors are essential because it has been widely shown how they can effectively improve several patient outcomes including medication adherence,^12^ hospital readmissions, depressive symptoms, quality of life,^13^ and symptom burden.^14,15^ Despite that, patients may experience difficulties in performing them^16–18^ because of various reasons such as older age, low self-efficacy, cognitive impairments, and comorbidities.^19,20^ In these cases, caregivers can play a critical role by contributing to patient self-care. Caregiver contribution to self-care is the process through which caregivers support patients in maintaining illness stability (ie, caregiver contribution to self-care maintenance), monitoring symptoms (ie, caregiver contribution to symptom monitoring and perception), and addressing symptoms (ie, caregiver contribution to self-care management).^21^

The presence of a chronic disease affects not only patients themselves but also their families and caregivers.^22^ Several theories and models, such as the Theory of Communal Coping,^23^ the Theory of Dyadic Illness Management,^24^ and the Heart Failure Care Dyadic Typology,^25^ explain how patients and their caregivers manage the chronic disease together, influence each other, and may find different coping strategies.^23,24^ Over the years, the Middle-Range Theory of Self-Care of Chronic Illness^10,11^ as well as the Situation-Specific Theory of Heart Failure Self-Care^26,27^ have been updated to account for symptom perception processes.^28^

Symptom perception includes cognitive processes of body awareness and body signal elaboration (ie, interoception)^29^ and involves complex interactions between cognition and behavior.^30^ Once a body signal or alteration is detected, it becomes possible to manage it.^10^ However, patients with a chronic disease, including HF, can experience difficulties in perceiving body changes and, thus, managing their symptoms.^29,31^ In such cases, caregivers can support patients in performing self-care (eg, in noticing body changes and managing symptoms).^15,27^ However, also caregivers may experience difficulties in contributing to self-care.^14,28^ Thus, there is a need to further understand how the members of the dyad are involved in self-care behaviors and how such involvement relates to symptom burden.

The Theory of Dyadic Illness Management^24^ stresses that the degree of congruence between patients' and caregivers' perceptions/responses to a chronic illness should be taken into account in any dyadic study, because it can influence the resulting individual and dyadic outcomes. However, studies doing so are scarce and, thus, warranted. Previous research explored dyadic behaviors focusing either on the dyad’s levels of symptom appraisal,^32^ or symptom management,^28,33^ or illness management.^34,35^ In HF, study authors often examine patients' and caregivers' experiences and outcomes separately, and only a minority of studies explore HF-related phenomena as they happen in the dyad as a team.^36^ In addition, although a few study authors explored the dyadic patterns of self-care behaviors in the dyad,^28,33,37^ there is a lack of knowledge on how dyadic congruence in self-care behaviors is related to the level of symptom burden in each dyad type.

Thus, in this study, we aimed to describe dyads in HF based on (*a*) the level of congruence between patient self-care and caregiver contribution to patient self-care and (*b*) the levels of patient symptom burden in each dyad type. This approach is broader compared with previous studies. Indeed, self-care theory is broader than symptom theory,^10^ and the symptom appraisal or management behaviors adopted by previous studies to identify dyadic types are themselves among the wider self-care behaviors. The results of this study could importantly support (*a*) the understanding how patient-caregiver dyads differently contribute to patient self-care and (*b*) the understanding of patients' levels of symptom burden depending on the dyadic self-care class they belong to. Ultimately, this could be pivotal to generating an evidence base for symptom management interventions depending on patient-caregiver dyad types.

## Methods

This is a secondary analysis of data collected in the MOTIVATE-HF randomized controlled trial,^38^ which is a randomized controlled trial primarily aimed at improving patient self-care through a motivational interviewing intervention. Briefly, patients with HF and their caregivers were recruited from 3 centers in Italy. Eligibility criteria for patients included being at least 18 years old, having HF^3^ with a New York Heart Association (NYHA) class II to IV, having insufficient self-care (ie, a score of 0, 1, or 2 on at least 2 items of the self-care maintenance or self-care management scales of the Self-Care of HF Index [SCHFI] v.6.2),^30^ and being willing to sign the informed consent form. Patients were excluded if they had a myocardial infarction in the last 3 months, had severe cognitive dysfunction, lived in a residential facility where self-care was not expected, or had an informal caregiver not willing to participate in the study. Eligibility criteria for caregivers were being identified by the patients as the primary caregiver. Participants were randomized among 3 arms. In arm 1, only patients received the intervention; in arm 2, both patients and caregivers received the intervention; and in arm 3, participants received standard care.^39^ For the present analysis, we only relied on baseline data. Data were collected between 2014 and 2018, after ethical approval for conducting the study was obtained by the Ethical Committee of the University of Rome Tor Vergata.

### Measurements

The authors of the MOTIVATE-HF study collected several outcomes through a battery of measurements.^39^ At baseline, when participants were found to qualify, a research assistant collected sociodemographic information and administered the questionnaires to both patients and caregivers. At follow-ups, data were collected telephonically. At both baseline and follow-ups, questionnaires were administered separately to patients and caregivers. For this study, we will only describe the main instruments used for the present analysis. Self-care was assessed using the SCHFI v.6.2,^40^ which is a valid and reliable instrument to measure self-care maintenance (ie, observing oneself for changes in signs and symptoms, and treatment adherence), management (ie, response to signs and symptoms when they occur), and confidence (ie, confidence that one has in the ability to perform a behavior). For each of the 3 components, 3 separate scores can be obtained, with ranges between 0 and 100. Higher scores indicate better self-care.

Caregiver contribution to self-care was assessed using the Caregiver Contribution to Self-Care of HF Index (CC-SCHFI),^41^ which is a valid and reliable instrument to measure caregiver contribution to patient self-care maintenance (ie, extent to which caregivers support patients in adhering to pharmacological and behavioral prescriptions and monitor symptoms), caregiver contribution to self-care management (ie, how likely caregivers are to help patients responding to their symptoms), and caregiver confidence (ie, caregiver self-efficacy in contributing to self-care). Also for the CC-SCHFI, 3 separate scores can be obtained (ranges between 0 and 100), with higher scores indicating better caregiver contribution to patient self-care.

Physical symptom burden was assessed using the Heart Failure Somatic Perception Scale^31^ (total score), which is a valid and reliable instrument^42^ to measure HF physical symptom burden and consists of 18 items grouped into 4 dimensions: chest discomfort, dyspnea, early and subtle symptoms, and edema. Each item can be rated from 0 to 5, with higher scores indicating higher symptom burden. Scores can range from 0 to 90, with higher scores indicating higher symptom burden.

To describe the sample, we relied on the following variables: age, gender, civil status, caregiver relationship with the patient, New York Heart Association class (indicating the severity of HF from I to IV), Charlson Comorbidity Index assessing comorbidities, number of medication, anxiety and depression (with the Hospital Anxiety and Depression Scale, with a total score ranging from 0 to 21 for each dimension; higher scores indicate higher anxiety/depression), mutuality (total score range from 0 to 4; higher scores indicate greater mutuality), physical and mental quality of life (with the Short Form 12; each scale score ranges from 0 to 100, and higher scores indicate better perceived health condition), and sleep quality (with the Pittsburgh Sleep Quality Index ranging from 0 to 21; higher scores indicate worse sleep quality).

### Statistical Analysis

Descriptive statistics, including means and standard deviations or count and percentages, were used to describe the overall sample. Latent class mixture modeling^43^ was used to identify distinct patterns among empirical Bayes estimates of within-dyad incongruence in self-care maintenance, management, and confidence as well as Heart Failure Somatic Perception Scale score. Dyadic incongruence (ie, second-order difference is patient and caregiver contributions to care adjusted for interdependence and measurement error)^44^ was calculated using fixed-effects dyadic models in Mplus v8 wherein the slope is the parameter that represents dyadic incongruence. Ram and Grimm criteria were used to compare latent class mixture models and identify the best solution (ie, lowest Bayesian information criteria, entropy closest to 1, classification probabilities closet to 1.0, significant Lo-Mendell-Rubin likelihood ratio test, and significant parametric bootstrap likelihood ratio test)—all generated as standard output in Mplus v8. χ^2^ Tests and analysis of variance were used to compare characteristics among the identified classes. An *α* of <.05 was considered significant. Descriptive and comparative statistics were generated using Stata/MP v18.

## Results

### Characteristics of the Sample

A total of 510 dyads of patients with HF and their caregivers were included. Patients were typically older adults (72.4 ± 12.3 years) and mainly partnered (62%) men (58%). Most of them had NYHA classes II to III (92.8%). Caregivers were 55 years old on average and mainly female (75.5%), married (72%), employed (73.5%), and living with the patients (60%).

### Dyadic Classes and Symptom Burden

We identified 3 classes based on the congruence between patient self-care and caregiver contributions to HF care in the dyads as well as patients' physical symptoms (Table 1, Figure) (Bayesian information criteria < alternative models; entropy, 0.71; classification probabilities exceed 0.8; Lo-Mendell-Rubin likelihood ratio test, *P* = .036; parametric bootstrap likelihood ratio test, *P* < .001; see Supplementary Table A [http://links.lww.com/JCN/A355] for additional metrics of the model). In class 1 (17%) (caregiver > patient and higher symptom burden), caregivers contributed more to HF care than patients, and patients in this class reported the highest level of symptom burden. In class 2 (7%) (patient > caregiver and lower symptom burden), patients performed more self-care than caregivers contribute to HF care, and patients in this class reported the lowest level of symptom burden. In class 3 (76%) (caregiver ~ patient), patients and caregivers contributed similarly to HF care. Table 1 shows average incongruence between patients' and caregivers' scores. Table 2 shows patients' and caregivers' characteristics in each class.

**TABLE 1.** Average Incongruence Between Patients' and Caregivers' Self-care Scores, and Patient Physical Symptom Burden Scores in Each Class

	Average Incongruence	Meaning
Class 1 Patient < caregiver N = 87 dyads	Self-care maintenance incongruence, −22.68 Self-care management incongruence, −28.28 Self-care confidence incongruence, −34.77 HFSPS: mean, 36.258	**▪** Caregivers perform more self-care than patients **▪** Higher symptom burden in patients
Class 2 Patient > caregiver N = 37 dyads	Self-care maintenance incongruence, 34.34 Self-care management incongruence, 25.67 Self-care confidence incongruence, 26.55 HFSPS: mean, 26.738	**▪** Patients perform more self-care than caregivers **▪** Lower symptom burden in patients
Class 3 Patient = caregiver N = 386 dyads	Self-care maintenance incongruence, −5.72 Self-care management incongruence, −4.53 Self-care confidence incongruence, −2.80 HFSPS: mean, 29.803	**▪** Patients and caregivers perform similar levels of self-care ▪ Mid-level symptom burden in patients

Abbreviation: HFSPS, Heart Failure Somatic Perception Scale.

**FIGURE.**
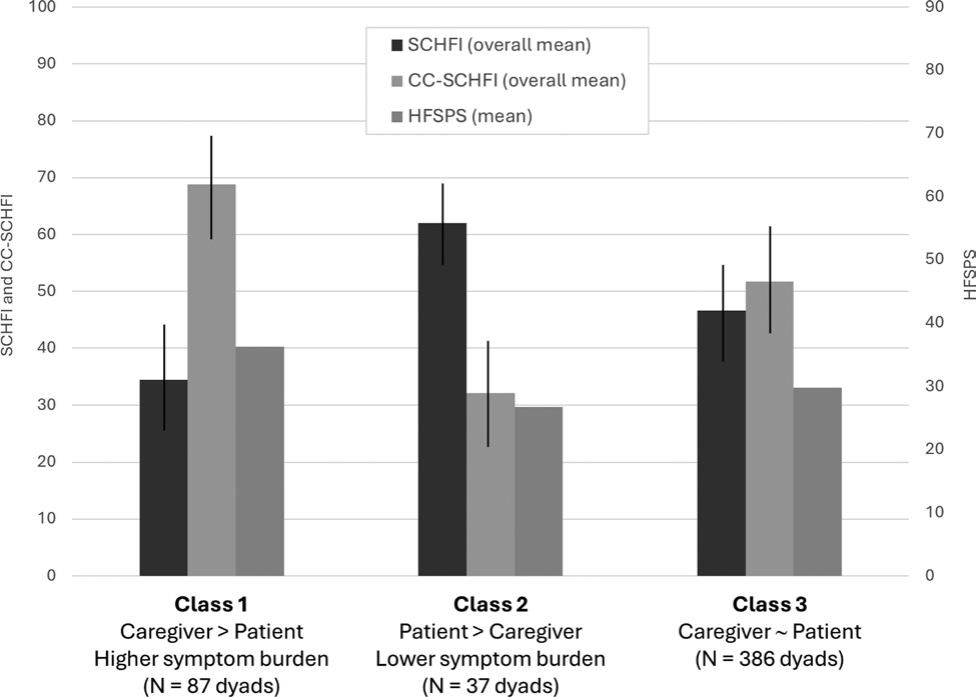
Graphical representation of the 3 identified classes. Mean overall scores for the SCHFI and CC-SCHFI have been computed only for graphical representations in this Figure to facilitate interpretability. For the analysis, as specified in the text and in Table 1, self-care dimensions have been scored separately. Metrics for this 3-class solution: BIC < alternative models; entropy, 0.71; classification probabilities exceed 0.8; LMRT, *P* = .036; PBLRT, *P* < .001. Abbreviations: CC-SCHFI, Caregiver Contribution to Self-Care of Heart Failure Index; HFSPS, Heart Failure Somatic Perception Scale; SCHFI, Self-Care of Heart Failure Index.

**TABLE 2:** Patients' and Caregivers' Characteristics in Each Class

Variable	Class 1 (n = 87 Pts, n = 87 Caregivers) Caregivers > Patients	Class 2 (n = 37 Pts, n = 34 Caregivers) Patients > Caregivers	Class 3 (n = 386 Pts, n = 382 Caregivers) Patients = Caregivers	Post Hoc (*P*)	χ^2^	ANOVA Between Groups, *P*
Patient age, y	75 (11)	68 (12)	72 (12)	1≠2 (.017)		.020
Caregiver age, y	52 (15)	57 (13)	54 (16)			
Patient gender	Male, 45 (52%) Female, 42 (48%)	Male, 21 (57%) Female, 16 (43%)	Male, 230 (60%) Female, 156 (40%)			
Caregiver gender	Male, 25 (29%) Female, 62 (71%)	Male, 14 (41%) Female, 20 (59%)	Male, 84 (22%) Female, 298 (78%)		0.026	
Patient’s civil status	44 (51%) married/cohabitant; 33 (38%) widow; 6 (7%) single; 4 (4.6%) divorced	25 (68%) married/cohabitant; 7 (19%) widow; 3 (8%) single; 7 (19%) divorced	247 (64%) married/cohabitant; 110 (28%) widow; 15 (4%) single; 14 (3.6%) divorced			
Caregiver’s civil status	61 (70%) married/cohabitant; 1 (1%) widow; 14 (16%) single; 11 (13%) divorced	23 (68%) married/cohabitant; 2 (6%) widow; 7 (20.6%) single; 2 (6%) divorced	277 (73%) married/cohabitant; 9 (2%) widow; 72 (19%) single; 23 (6%) divorced			
Crg’s relationship with the pt	Spouse, 28%; child, 46%	Spouse, 47%; child, 29%	Spouse, 39%; child, 38%		0.016	
Crg living with the pt	62% yes	62% yes	61% yes			
NYHA class	II, 47%; III, 47%; IV, 6%	II, 56%; III, 33%; IV, 11%	II, 66%; III, 28%; IV, 6%		0.009	
Charlson Comorbidity index	3.53 (2.34)	2.24 (1.36)	2.84 (1.91)	1≠2 (.001); 1≠3 (.031); 2≠3 (.047)		.001
HADS_Anxiety_Pt	8.41 (4.29)	7.05 (4.45)	7.75 (4.41)			
HADS_Depression_Pt	9.06 (4.06)	7.03 (4.79)	7.81 (4.42)	1≠2 (.057); 1≠3 (.050)		.023
HADS_Anxiety_crg	7.44 (4.45)	6.94 (4.17)	7.54 (4.56)			
HADS_Depression_crg	5.49 (3.90)	6.41 (4.85)	5.96 (4.43)			
No. medications (SD)	6.51 (2.66)	5.83 (2.71)	6.75 (2.96)			
Mutuality_pt	3.03 (0.62)	2.95 (0.65)	2.91 (0.61)			
Mutuality_crg	3 (0.61)	2.8 (0.66)	2.77 (0.67)	1≠3 (.011)		.015
SF Physical QoL_pt	32.43 (8.7)	38.43 (11.8)	35.85 (9.37)	1≠2 (.004); 1≠3 (.007)		.001
SF Mental QoL_pt	41.7 (10.56)	46.78 (11.00)	45.23 (9.88)	1≠2 (.031); 1≠3 (.010)		.006
SF Physical QoL_crg	48.50 (8.80)	46.15 (10.35)	49.02 (7.92)			
SF Mental QoL_crg	48.24 (9.81)	47.43 (10.05)	48.60 (9.23)			
PSQI_pt	12.88 (3.76)	12.00 (3.75)	12.20 (3.65)			
PSQI_crg	10.05 (3.58)	10.53 (3.68)	9.76 (3.27)			
HFSPS	36.258 (19.70)	26.738 (20.08)	29.803 (17.70)	1≠2 (.005) 1≠3 (.002) 2≠3 (.691)		.001
SCHFI-Maintenance	36.50 (17.19)	58.73 (14.70)	46.32 (13.83)	1≠2 (.000) 1≠3 (.000) 2≠3 (.000)		.000
SCHFI-Management	31.74 (18.84)	57.60 (15.06)	40.16 (16.29)	1≠2 (.000) 1≠3 (.000) 2≠3 (.000)		.000
SCHFI-Confidence	35.36 (20.37)	69.72 (16.02)	53.23 (20.15)	1≠2 (.000) 1≠3 (.000) 2≠3 (.000)		.000
CC-SCHFI-Maintenance	62.60 (19.05)	20.98 (14.60)	51.66 (17.31)	1≠2 (.000) 1≠3 (.000) 2≠3 (.000)		.000
CC-SCHFI-Management	67.09 (18.15)	35.33 (17.47)	47.92 (18.80)	1≠2 (.000) 1≠3 (.000) 2≠3 (.034)		.000
CC-SCHFI-Confidence	76.74 (17.88)	40.06 (19.87)	55.52 (21.25)	1≠2 (.000) 1≠3 (.000) 2≠3 (.000)		.000

Data are presented as mean (SD). In the case of age, gender, civil status, relationship with the patient/caregiver, and NYHA class, data are presented as percentages or number (%). Bonferroni test was used for post hoc analysis because the assumption of homogeneity of variance was respected. Higher scores indicate greater levels of the dimension being measured, apart from PSQI where higher scores indicate poorer sleep. Only statistically significant differences are reported in the table.

Abbreviations: ANOVA, analysis of variance; CC-SCHFI, Caregiver Contribution to Self-Care of Heart Failure Index; Crg, caregiver; HADS, Hospital Anxiety and Depression Scale; HFSPS, Heart Failure Somatic Perception Scale; NYHA, New York Heart Association; PSQI, Pittsburgh Sleep Quality Index; Pt/pt, patient; QoL, Quality of Life; SCHFI, Self-Care of Heart Failure Index; SF, Short Form.

In class 1, where caregivers were contributing more to self-care, they were the youngest caregivers and patients in this class were the oldest compared with the other classes. In this class, caregivers were mainly the children of the patients (46%), whereas in the other classes, caregivers were often the spouse of the patients. In general, patients in class 1 were more compromised compared with the patients in the other classes. Indeed, patients in class 1 were either in NYHA class II or III (47% and 47%, respectively), whereas patients in the other classes were mainly in NYHA class II; they had more comorbidities, higher levels of anxiety and depression, and lower mental and physical quality of life. Both patients and caregivers in class 1 reported higher levels of perceived mutuality between them compared with the dyads in the other classes.

Contrarily, in class 2, where patients were performing more self-care than caregivers, patients were younger and caregivers were older compared with the other classes (although the difference was not always significant). In this class, patients reported the lowest comorbidity, the lowest anxiety and depression, and the highest mental and physical quality of life. Furthermore, patients in this class were taking less medications than patients in the other classes and were mainly (56%) in NYHA class II. On the other hand, caregivers in class 2 were predominantly (47%) the patient’s spouse and tended to have greater anxiety and depression as well as lower physical and mental quality of life compared with caregivers in the other classes (although the difference was not always significant).

Dyads in class 3 showed average scores in most of the variables being measured, locating in between the scores of dyads in classes 1 and 2. Caregivers in class 3 were equally distributed between being the spouse and the child of the patient (39% and 38%, respectively) and (although not statistically significantly different) reported higher mental quality of life and better sleep compared with caregivers in the other classes. Patients in this class were predominantly in NYHA class II (66%) and were taking slightly more medications than patients in the other classes.

## Discussion

In this study, we aimed to explore (*a*) whether self-care behaviors are performed differently by patients and caregivers in the same dyad and (*b*) the levels of symptom burden in each dyad type. We identified 3 classes based on the average congruence between patient self-care and caregiver contribution to patient self-care in the dyads. Specifically, we identified a class of dyads where caregivers contributed to self-care more than patients, and patients in this class had the highest level of symptom burden; another class where patients performed more self-care than caregivers, and patients in this class had the lowest level of symptom burden; and another class where patients and caregivers equally contributed to self-care. These classes we observed align with those previously described by the Heart Failure Care Dyadic Typology^25^: the patient-oriented, caregiver-oriented, and collaborative-oriented dyadic care types.

In class 1, where caregivers contributed to self-care more than patients, patients reported the highest level of symptom burden. This highlights the concurrent presence of higher caregiver contribution to self-care and higher patient symptom burden, when patients were not engaged. Ultimately, this could suggest that patient engagement in self-care behaviors might be one important factor to observe a concurrent lower symptom burden. This highlights that patients belonging to this class might need heightened support to increase their engagement in self-care behaviors and to report a lower level of symptom burden. This is further supported by the finding that patients in class 2 were contributing more to self-care compared with their caregivers and reported the lowest levels of symptom burden compared with patients in the other classes. Previous longitudinal studies found that when patients with HF performed more self-care, they reported less symptom burden.^15^ In that case, results also showed that more caregiver contribution to self-care was associated with more patient self-care, which, in turn, was associated with lower symptom burden. In this cross-sectional study, the results suggest that, even when caregivers contribute more to self-care, patients do not necessarily engage much in self-care and still concurrently report higher symptom burden than patients in the other classes. This suggests that it might also depend in which stage of contribution to self-care the dyad is. For example, it could be that caregivers may need to contribute very much to patient self-care to have patients actually performing more self-care and better control their symptom burden. Moreover, the results of the previously mentioned study^15^ referred to the dimension of self-care maintenance, but the results did not hold for self-care management.

We also found that patients in class 1 (where caregivers contributed more to self-care compared with patients) were the oldest, had more comorbidities, and had the lowest physical quality of life. This may explain why caregivers intervened more in patient self-care practices. We also found that patients in this class were also those with the highest levels of depression (and anxiety, although not fully significant) and the lowest mental quality of life. This is consistent with previous literature^45^ highlighting that patients who are depressed, anxious, or cognitively impaired find it difficult to perform self-care (eg, remembering recommendations, monitoring and interpreting symptoms) and benefit less from its practice. Brain structural changes involved in emotional and cognitive functions have been documented in patients with HF,^46,47^ and this might explain why depression, mental quality of life, and low self-care practices (including symptom monitoring and management) occur as a class and together with higher symptom burden. This further highlights that patients belonging to this class might need heightened support in managing their condition and adopting effective self-care behaviors. On the other hand, our findings showed that patients in class 2 were in the most favorable conditions compared with patients in the other classes, whereas their caregivers tended toward worse conditions compared with those in the other classes (eg, greater anxiety and depression, lower physical and mental quality of life). Although patients in this class seem to adopt greater self-care behaviors and experience lower symptom burden, our findings might suggest that caregivers belonging to this class should not be disregarded but, instead, supported in their caregiving role not to risk that their condition worsen and lead them to unfavorable outcomes.

Symptom science and self-care science are intrinsically related,^10^ as the processes of self-care monitoring and management imply perceiving and responding to symptoms and symptoms exert a strong influence on the self-care decision-making process.^10^ For instance, people may be more willing to engage in self-care behaviors if they have symptoms, but depressive symptoms and cognitive deficits can also decrease motivation to engage in self-care behaviors.^48,49^ At the same time, self-care behaviors can also influence the symptom burden, and the symptom frequency and intensity.^50^ Understanding the levels of symptom burden is crucial to facilitate HF management, and self-care plays a crucial role in addressing symptoms appropriately.^15^ Considering that self-care is a dyadic phenomenon, in which patients and caregivers can interact and contribute to self-care in different ways and combinations,^24,33^ the results of this study are crucial because they provide insights into the interplay between dyadic self-care and symptoms; more precisely, they show how symptom burden varies across the different classes of dyadic self-care congruence. These results, indeed, could help tailoring interventions to support self-care and symptom management in the dyads by finding a balance between individualized and standardized interventions. Authors of future studies could longitudinally explore how these dyadic patterns evolve over time, maybe also including patients more equally distributed between the 4 NYHA classes. In addition, future research could build on the insights provided by this study by developing tailored interventions to optimize symptom management. For example, authors of future studies could examine how digital tools can enhance patient-caregiver collaboration in HF management. Telemonitoring systems and mobile health applications could facilitate real-time communication, improve shared decision making, and support self-care and symptom management. Investigating their impact could help develop effective technology-assisted dyadic care strategies to optimize HF outcomes.

### Strengths and Limitations

Patients in our sample had poor self-care and were mainly in NYHA classes II and III, which might reduce the generalizability of the results. However, we recruited patients from 3 different settings, which may compensate for that limitation and enhance the generalizability of results across different settings. Our approach to latent class mixture modeling is based on conventional parameters to assess strengths and weaknesses, and our model entropy was indicative of some uncertainty in dyad classification. As such, future research in HF dyads may reveal slightly different classes. Ultimately, we would want to know how dyads change over time but are limited in our understanding of stability, among other levels of inference, given the cross-sectional nature of these data.

## Conclusion

The results of this study show that there are different dyads of patients with HF and their caregivers depending on the congruence between their levels of self-care and contribution to self-care, respectively. In some dyads, patients perform more self-care compared with how much their caregivers contribute to self-care, and in these dyads, patients experience the lowest level of symptom burden. In other dyads, caregivers contribute more to self-care compared with how much patients themselves perform self-care, and in these dyads, patients experience the highest level of symptom burden. This is pivotal to appreciating how patient-caregiver dyads differently contribute to patient self-care and to understanding patients' levels of symptom burden depending on the dyadic self-care class they belong to. Ultimately, this can help generate an evidence base for symptom management interventions depending on patient-caregiver dyad types.

## Supplementary Material


