# Supplementary material for: Profiles of Dyadic Self-care Congruence and Patient Symptom Burden in Heart Failure
Source: J Cardiovasc Nurs. 2025 Aug 11;41(2):104–10. doi: 10.1097/JCN.0000000000001239 (PMC12875626; doi:10.1097/JCN.0000000000001239)
Supplement: Supplementary file 1 [file jcn-41-104-s001.docx]

**Supplementary file**

**Table A.** Other metrics of the MLC analysis

| Model | BIC | Entropy | Classification Probabilities | LMRLRT | PBLRT |
| --- | --- | --- | --- | --- | --- |
| 2 | 17169 | 0.73 | 0.94/0.81 | 87.2, p=0.042 | p<0.001 |
| **3** | **17124** | **0.71** | **0.81/0.85/0.88** | **58.4, p=0.039** | **p<0.001** |
| 4 | 17114 | 0.72 | 0.82/0.78/0.86/0.87 | 24.8, p=0.183 | p<0.001 |
| 5 | 17101 | 0.70 | 0.92/0.82/0.80/0.78/0.85 | 27.3, p=0.279 | p<0.001 |
| 6 | 17089 | 0.71 | 0.87/0.86/0.83/0.79/0.77/0.86 | 26.5, p=0.204 | p<0.001 |

**Abbreviations**: BIC = Bayesian Information Criteria; LMRLRT = Lo-Mendel-Rubin Adjusted Likelihood Ratio Test; PBLRT = Parametric Bootstrap Likelihood Ratio Test. **Note**: We reported in bold the model we eventually chose.
